# Integrated transcriptomic and metabolomic analyses reveals anthocyanin biosynthesis in leaf coloration of quinoa (*Chenopodium quinoa* Willd.)

**DOI:** 10.1186/s12870-024-04821-2

**Published:** 2024-03-20

**Authors:** Min Zhang, Yueyou Li, Junling Wang, Shaopu Shang, Hongxia Wang, Xinlei Yang, Chuan Lu, Mei Wang, Xinbo Sun, Xiaoqing Liu, Xiaoxia Wang, Boxiang Wei, Wei Lv, Guojun Mu

**Affiliations:** 1 North China Key Laboratory for Crop Germplasm Resources of Education Ministry, The Key Laboratory of Germplasm Resources of Hebei Province, Hebei Agricultural University, Baoding, Hebei Province 071000 P. R. China; 2 The Quinoa Industrial Technology Research Institute of Hebei Province, Zhangjiakou, Hebei Province 075000 P. R. China; 3 The Quinoa S&T Academy Park of Rural Special Technology Association of China, Zhangjiakou, Hebei Province 075000 P. R. China; 4 The S&T Innovation Service Center of Hebei Province, Shijiazhuang, Hebei Province 050000 P. R. China

**Keywords:** DEGs, DAMs, TFs, Integrated transcriptomic and metabolomic analyses, qRT-PCR

## Abstract

**Background:**

Quinoa leaves demonstrate a diverse array of colors, offering a potential enhancement to landscape aesthetics and the development of leisure-oriented sightseeing agriculture in semi-arid regions. This study utilized integrated transcriptomic and metabolomic analyses to investigate the mechanisms underlying anthocyanin synthesis in both emerald green and pink quinoa leaves.

**Results:**

Integrated transcriptomic and metabolomic analyses indicated that both flavonoid biosynthesis pathway (ko00941) and anthocyanin biosynthesis pathway (ko00942) were significantly associated with anthocyanin biosynthesis. Differentially expressed genes (DEGs) and differentially accumulated metabolites (DAMs) were analyzed between the two germplasms during different developmental periods. Ten DEGs were verified using qRT-PCR, and the results were consistent with those of the transcriptomic sequencing. The elevated expression of phenylalanine ammonia-lyase (*PAL*), chalcone synthase (*CHS*), 4-coumarate CoA ligase (*4CL*) and Hydroxycinnamoyltransferase (*HCT*), as well as the reduced expression of flavanone 3-hydroxylase (*F3H*) and Flavonol synthase (*FLS*), likely cause pink leaf formation. In addition, *bHLH14*, *WRKY46*, and *TGA* indirectly affected the activities of *CHS* and *4CL*, collectively regulating the levels of cyanidin 3-O-(3*’’*, 6*’’*-O-dimalonyl) glucoside and naringenin. The diminished expression of *PAL*, *4CL*, and *HCT* decreased the formation of cyanidin-3-O-(6”-O-malonyl-2”-O-glucuronyl) glucoside, leading to the emergence of emerald green leaves. Moreover, the lowered expression of *TGA* and *WRKY46* indirectly regulated *4CL* activity, serving as another important factor in maintaining the emerald green hue in leaves N1, N2, and N3.

**Conclusion:**

These findings establish a foundation for elucidating the molecular regulatory mechanisms governing anthocyanin biosynthesis in quinoa leaves, and also provide some theoretical basis for the development of leisure and sightseeing agriculture.

**Supplementary Information:**

The online version contains supplementary material available at 10.1186/s12870-024-04821-2.

## Background

Quinoa (*Chenopodium quinoa* Willd., 2n = 4x = 36), recognized as the “king of food”, “nutritional gold”, “food of the future”, and “super food”, originated in the Andes mountains of South America [1]. This remarkable grain has garnered global attention because of its exceptional nutritional profile, health benefits, and robust resistance to adverse environments [2]. Quinoa has immense potential in various sectors, including food, medicine, livestock feed, and industry [3]. It is abundant in essential nutrients, such as vitamins, polyphenols, flavonoids, saponins, phytosterols, and other bioactive compounds, offering high protein content, rich dietary fiber, easy digestibility, and a low predicted glycemic index [4].

Quinoa, esteemed for its high ornamental value, serves a dual purpose as both a food crop and a contributor to landscape greening, enhancing the development of leisure and sightseeing agriculture [5]. Quinoa seedlings initially display emerald green leaves. As quinoa ripens, its leaves gradually develop a range of vibrant colors, such as pink, green, yellow, purple, red, and orange, contributing to the creation of an aesthetically pleasing landscape [6]. The diversity in leaf coloration is closely related to anthocyanin biosynthesis. Anthocyanins, a type of flavonoid, are widespread in various plant leaves, fruits, and other tissues and organs and are known for their potent antioxidant properties [7, 8]. They are soluble in water, methanol, and other solvents [9] and can inhibit cancer cell growth [10], inflammation, and obesity [11], while also promoting vascular health [12], preserving eyesight, and contributing to beauty care.

The synthesis and metabolism of anthocyanins in quinoa is a complex, yet well-defined biosynthetic pathway. Zheng et al. [13] observed significant up-regulation of flavanone 3-hydroxylase (*F3H*), flavonoid 3’-5’ hydroxylase (*F3’5’H*), and dihydroflavonol reductase (*DFR*), along with notable down-regulation of flavonol synthase (*FLS*), in red and purple flowers compared with white flowers of *Nicotiana alata*. Luo et al. [14] reported lower expression levels of phenylalanine ammonia-lyase (*PAL*), chalcone synthase (*CHS*), and chalcone isomerase (*CHI*) in green shamrock flowers than in purple shamrock flowers. In the case of tea (*Camellia sinensis* L.), *DFR* was up-regulated in pink flowers relative to white flowers, whereas *FLS* displayed higher expression levels in white flowers [15]. In addition to these structural genes, several regulatory genes can affect the anthocyanin synthesis pathway. These genes operate indirectly by acting as transcription factors (TFs), thereby modulating the expression of structural genes. At the transcriptional level, the TF family governing anthocyanin biosynthesis predominantly comprises the v-myb avian myeloblastosis viral oncogene homolog (MYB), basic helix-loop-helix (bHLH), and WD40, which form complexes that directly bind to the promoters of anthocyanin biosynthesis structural genes [16]. Nesi [17] and Baudry et al. [18] demonstrated that TESTA 2 (TT2) (MYB) can regulate late biosynthesis genes by binding to TT8 (bHLH42) and TTG1 (WD40), resulting in seed coat pigmentation. Yang et al. [19] found the repression of R2R3 MYB TF in white petals compared to pink petals. Meng et al. [20] found that *MrMYB44-like1*, *MrMYB44-like2*, and *MrMYB44-like3* of the MYB TF family were highly expressed in green leaves of *Malus* crabapple (*Malus Mill*.). Jin et al. [21] identified that MYB28.1 and RL1 are associated with purple pigmentation of purple/green leaf. Additionally, Song et al. [22] discovered that MYB2 plays a positive regulatory role in determining the purple trait in purple head Chinese cabbage (*Brassica rapa* L.).

The molecular regulatory mechanisms governing leaf coloration in quinoa remain unexplored. In this study, germplasm materials exhibiting emerald and pink leaf hues were selected from the germplasm resources of quinoa at the Science and Technology Innovation Service Center in Hebei Province, China. Employing an integrated approach of transcriptomics and metabolomics, DEGs and DAMs were investigated for their association with anthocyanin biosynthesis in different leaf colors. The results of this study are anticipated to provide pivotal insights for comprehensive exploration of the molecular mechanisms underlying anthocyanin biosynthesis in quinoa.

## Materials and methods

### Plant materials

The examined quinoa varieties, M202 and M146, displayed emerald green and pink leaves, respectively. These varieties were cultivated at the Zhangbei Experimental Station of Hebei Agricultural University on May 25, 2021 and May 26, 2022, with row spacing of 40 cm and plant spacing of 18 cm.

Leaf characteristics of M202 and M146 were recorded from 20 to 110 d of growth, with observations at 10-d intervals. The leaves from the middle of each plant were selected for evaluation. Three distinct stages were identified: color change initiation (70 days after germination, 70 DAG), partial color change (90 DAG), and full color change (110 DAG). The stages were labeled as N1, F1, N2, F2, and N3, F3 for M202 and M146, respectively, representing the initial, elongation, and maturity stages of leaf development. Each sample, ranging from 1 to 3 g, was used for three biological replicates. Samples were sealed in plastic bags with 5–10 grains of silica gel and stored at -80℃.

### Identification of leaf color in quinoa

Samples from stages N1, N2, N3, F1, F2, and F3 were quantified using a chromatic meter (CR-10plus, Japan) in triplicate. “L” represents black and white value, “+”and “–” represent partial white and duskiness; “a” represents the red-green value, “+”and “–” represent reddish and green, respectively; “b” indicates the yellow-blue value, “+” and “–” indicate yellowish and bluish, respectively. Based on the criteria established for N1 and F1, difference calculations (ΔE=[(ΔL)^2^+(Δa)^2^+(Δb)^2^]^1/2^) were conducted using SPSS26. When ΔE ≤ 2, the color difference is imperceptible to the naked eye; when 2 < ΔE ≤ 6, the color difference can be discerned but is not pronounced; and when ΔE *>* 6, the human eye can easily distinguish the color difference.

### Quantification of total anthocyanin in quinoa leaves

The anthocyanin content was assessed using the method described by Serrano [23]. Each 0.5 g sample (N1, F1, N2, F2, N3, and F3) was ground into powder in 0.1 mol/L hydrochloric methanol solution. The mixture was then shielded from light in tin foil and maintained at 4℃ until the tissue had a white appearance. Subsequently, centrifugation at 10,000 *g* for 10 min was performed, and the absorbance of the supernatant was measured at 530 and 657 nm. The difference in anthocyanin absorbance at 530 nm was calculated using the equation A_530_-0.25×A_6__57_. This value was normalized to the fresh weight to represent the total anthocyanin content. The total anthocyanin content of each sample was statistically analyzed by hypothesis test. *P* > 0.05 indicated no significant difference. 0.01 < *P* < 0.05 indicated significant difference and marked with *. *P* < 0.01 and marked with **.

$${\rm{Anthocyanin}}\,{\rm{content}}\,\left( {{\rm{OD}}\,{\rm{/}}\,{\rm{g}}} \right)\, = {\mkern 1mu} {\rm{(}}{A_{{\rm{530}}}}\, - \,{\rm{00}}{\rm{.25}}\, \times \,{A_{{\rm{657}}}}{\rm{)}}\,{\rm{/}}\,{\rm{m}}$$

*A530*: Absorption value of anthocyanin at 530 nm.

*A657*: Absorption value of anthocyanin at 657 nm.

*m*: weight of sample (unit: g).

### Transcriptomic sequencing indicated extremely significant difference

RNA was extracted from the samples using the TRIzol precipitation method in Rio [24]. Subsequently, a cDNA library was prepared and the library check was completed. The samples were sequenced on the Illumina (http://www.illumina.com) platform with three biological replicates. The basic group mass values for the raw data were determined using Sequencing by Synthesis (SBS) techniques. High-quality clean data were obtained by removing reads containing joints, reads with an N ratio exceeding 10%, and reads with both a mass value Q ≤ 10 and a base number surpassing 50% of the entire read.

High-quality clean data were compared to the tetraploid quinoa reference genome (https://www.ncbi.nlm.nih.gov/datasets/taxonomy/63459/) using an efficient HISAT2 [25] comparison system. Subsequently, StringTie [26] was employed for the assembly and quantification of the reads, generating the initial transcript. This transcript was further compared with various databases, including Nr (https://ftp.ncbi.nih.gov/blast/db/), Swiss-Prot (http://www.uniprot.org/), GO (http://www.geneontology.org/), and KEGG (http://www.genome.jp/kegg/) using BLAST. Annotation information was obtained by employing the HMMER [27] software in conjunction with the Pfam database.

#### Identification of DEGs

The transcript expression levels were normalized for accurate representation. StringTie [28] employed Fragments Per Kilobase Million (FPKM) as a metric to quantify transcript or gene expression levels using a maximum traffic algorithm. DESeq2 [29] software facilitated the analysis of DEGs. DEGs were selected based on the following criteria: |log2(FC)| ≥ 1 and False Discovery Rate (FDR) < 0.05, with Fold Change (FC) indicating differences in gene expression levels. DEGs were classified as up-regulated (log2(FC) > 1) or down-regulated (log2(FC) < 1). R software was used to generate volcano plots, visually representing the DEGs statistics for each comparison group compared to the FDR values. Gene overlap analyses were conducted for the selected DEGs and Venn diagrams were constructed to illustrate the unique and shared genes among the comparison groups.

#### GO and KEGG enrichment analyses of DEGs

Functional annotation of DEGs was performed using GO and KEGG databases. GO enrichment analysis was conducted using the GO database (http://www.geneontology.org/), which includes three distinct ontologies: molecular function (MF), cellular component (CC), and biological process (BP) [30]. KEGG enrichment analysis was conducted using the KEGG (http://www.genome.jp/kegg/). The DEGs were systematically associated with terms in the GO database to calculate the number of genes within each GO terms. Hypergeometric tests were employed to identify the significant enrichment of GO terms within the DEGs in contrast to the entire genome background. In KEGG enrichment analysis, pathways were treated as the units of analysis. The hypergeometric test was applied to identify pathways exhibiting significant enrichment in the DEGs relative to the background gene set. For both GO and KEGG analyses, pathways were considered significantly enriched if they yielded a q-value (Benjamini - Hochberg-corrected p-value) of less than 0.05.

### Metabolomic analysis

The metabolites were extracted and isolated according to the method of Huan et al. [31]. Metabolites were analyzed using liquid chromatography-tandem mass spectrometry (LC-MS/MS) for both qualitative and quantitative assessments. Original data from the mass spectrometry analysis underwent baseline filtering. Peak identification, filtering, and alignment of each metabolite were performed using the XCMS package (v3.3.2) in R. This process yielded a data matrix of the mass-to-charge ratio (m/z), retention time, and peak intensity. Finally, the filtered data from all platforms were normalized based on the weight of samples used for extraction and according to sample median intensity group-wise, and the resulting data matrices were utilized for further analysis. Metabolite annotations were determined by referencing public mass spectrometry databases such as HMDB (http://www.hmdb.ca/), Massbank (http://www.massbank.jp/), LipidMaps (http://www.lipidmaps.org), and METLIN [32] (http://metlin.scripps.edu/index.php).

All detected metabolites were annotated using the MetWare database. Metabolomic data were subjected to multivariate statistical analysis using MetaboAnalyst software (version 5.0). Unsupervised dimension reduction and principal component analysis were performed using the R package (http://www.r-project.org/) to visualize distinctions among the sample groups. Additionally, the orthogonal projection to latent structures-discriminant analysis (OPLS-DA) model was rigorously validated using cross-validation and permutation tests. Score and permutation plots were generated to illustrate the differences between comparison groups. Variable importance in projection (VIP) scores were used to describe the overall contribution of each variable to the model. The criteria for identifying DAMs were set at VIP > 1, |log2(FC)| ≥ 1, and *P* < 0.05.

### Integrated transcriptomic and metabolomic analyses

The combined analysis of transcriptomic and metabolomic is based on the standard analysis results obtained from both omics approaches. By analysing the transcriptomics and metabolomics results, the simultaneous mapping of DEGs and DAMs binding onto the KEGG pathway map allows for a better explanation of the transcriptional regulatory mechanisms in metabolic pathways. DEGs and DAMs were subjected to joint analysis using the Pearson correlation coefficient (PCC) method. The threshold of PCC ≥ 0.6 and *P* < 0.05 was applied for this analysis. PCC ≥ 0.6 indicates a significant correlation between DEGs and DAMs.

### Analysis of differentially expressed TFs

The selected DEGs were subjected to BLAST comparisons with the quinoa genome database and were subsequently combined with the PlantTFDB database (http://planttfdb.cbi.pku.edu.cn/) to identify differentially expressed TF genes. These selected TFs, along with their genes associated with anthocyanin biosynthesis, were further analyzed. The expression patterns of these differentially expressed TFs were analyzed according to the FPKM values from the transcriptomic data.

### qRT-PCR verification

RNA was extracted and subjected to reverse transcription to synthesize cDNA for qRT-PCR verification. The reaction conditions comprised denaturation at 95℃ for 10 s, annealing at 60℃ for 20 s, and extension at 72℃ for 15 s for a total of 40 cycles. Using *ACT7* [33] as an internal reference, primers were designed using Premier5.0 software based on the CDS sequence corresponding to the target gene in the sequence library (Supplementary Table 1). Relative expression levels were determined using the 2^−ΔΔCt^ method [34]. Three biological replicates were performed for each qRT-PCR reaction.

## Results and analysis

### Phenotypic observation of leaf color in quinoa

**Fig. 1:**
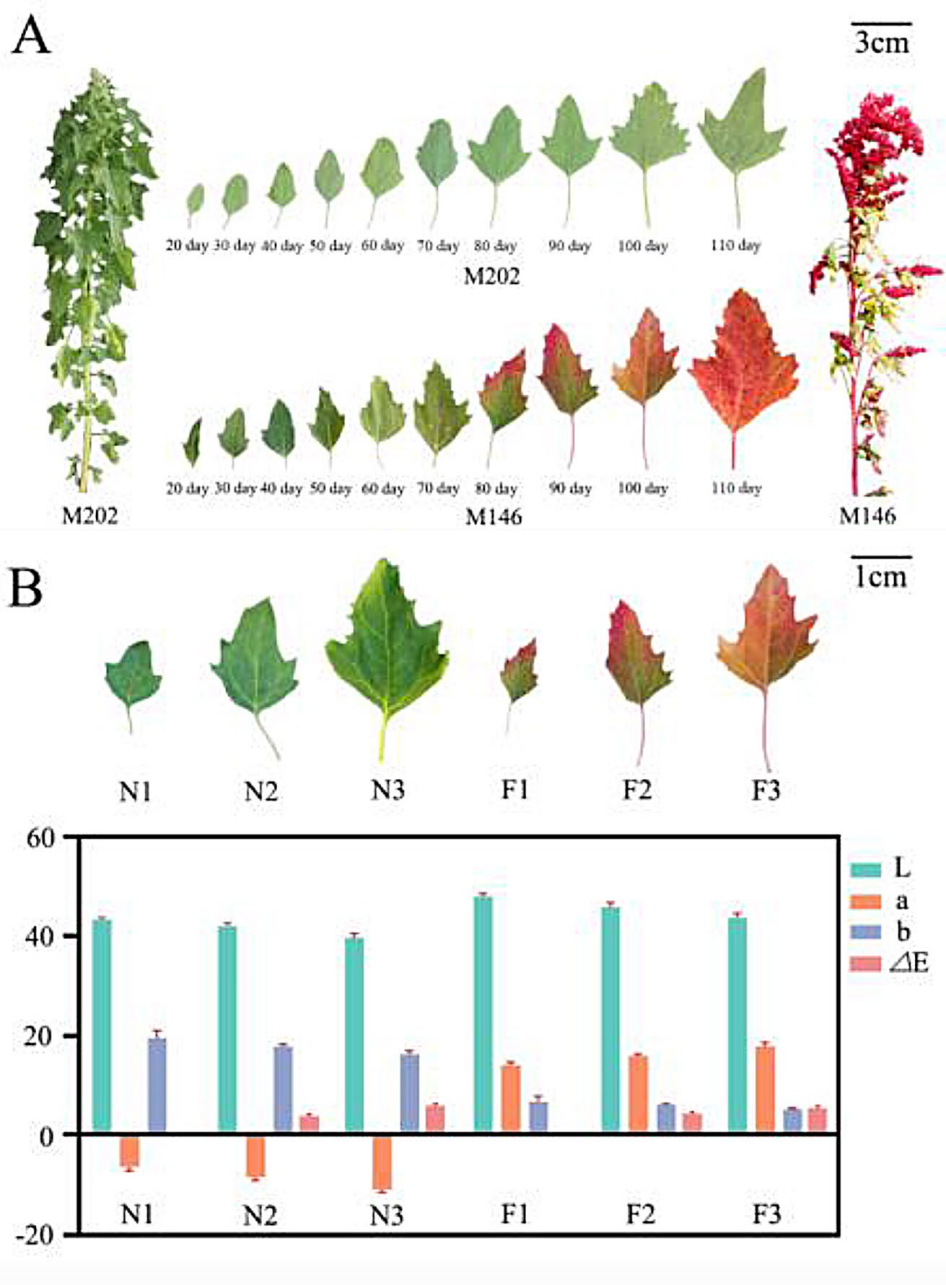
Different stages of leaf development and values of chromatism between two quinoa varieties. (**A**): Growth changes of quinoa leaves from 20 to 110 d and plant phenotypes at maturity (**B**): Color difference values (L, a, b, and ΔE) for six samples

The growth changes in quinoa leaves, spanning from 20 to 110 d, and the phenotypes at maturity were recorded (Fig. 1A). Leaves at 70, 90, and 110 d represented the stages of color transition, half coloration, and full coloration, respectively. The colorimeter readings for M202 revealed L values between 38 and 44, a values between − 12 and − 5, and b values between 15 and 22 (Fig. 1B). Moreover, M146 exhibited L values between 42 and 49, a values between 13 and 19, and b values between 4 and 8. Both M202 and M146 displayed darkening of leaf color as the developmental period was extended. The ΔE values, indicating color differences, increased with leaf growth. When N1 was the reference, ΔE values for N2 and N3 were 3.13 and 6.87, respectively. Similarly, using F1 as the standard, ΔE values for F2 and F3 were 2.87 and 5.95, respectively.

### Quantification of total anthocyanin content

**Fig. 2:**
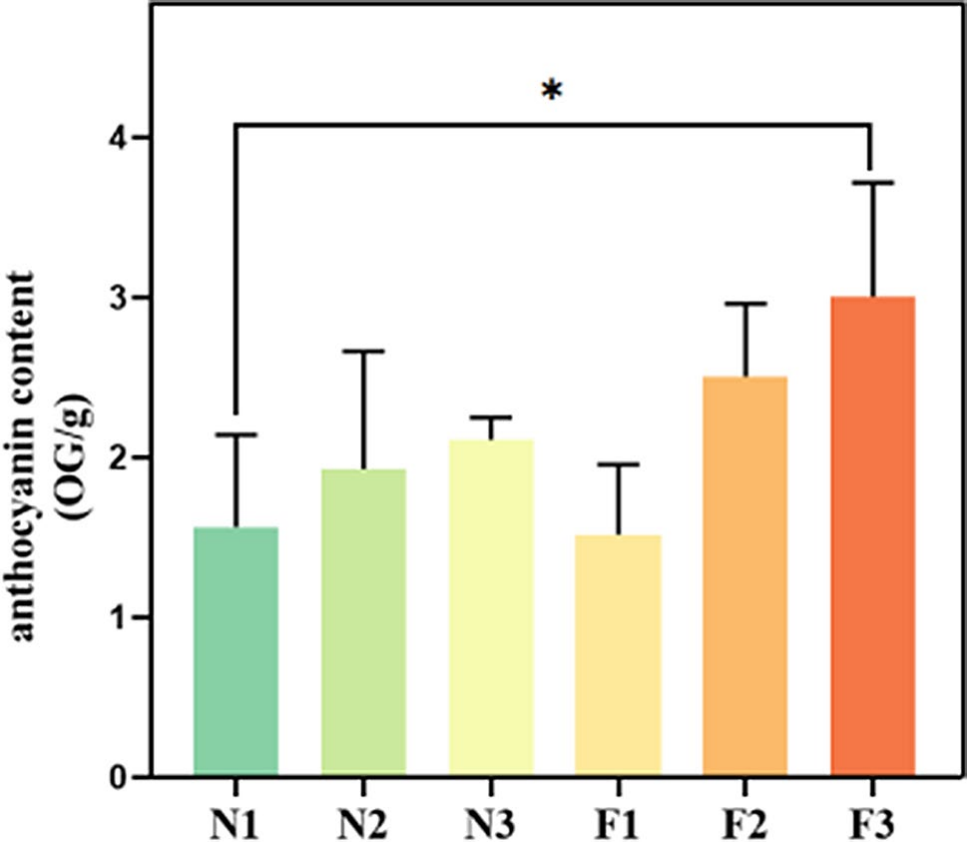
Changes in anthocyanin content among different quinoa varieties and leaf developmental periods. The vertical axis represents anthocyanin content (OG/g), the abscissa indicates the name of each sample, and * indicates statistically significant differences (*p* < 0.05)

Quantification analysis revealed a progressive increase in the total anthocyanin content in quinoa leaves as they matured. Specifically, at stages N1, N2, N3, F1, F2, and F3, the total anthocyanin content in the leaves was 1.57, 1.93, 2.11, 1.52, 2.50, and 3.00 OG/g, respectively (Fig. 2).

### Transcriptomic analysis and screening of DEGs

Eighteen cDNA libraries were generated from the leaf samples at stages N1, F1, N2, F2, N3, and F3. Transcriptomic sequences were obtained after the removal of adapters and low-quality reads. A total of 140.52 Gb of high-quality clean data were obtained, with a Q20 base composition percentage exceeding 97.52% and a Q30 base composition percentage exceeding 92.96%. The GC content measured greater than 45.15% (Supplementary Table 2).

**Fig. 3:**
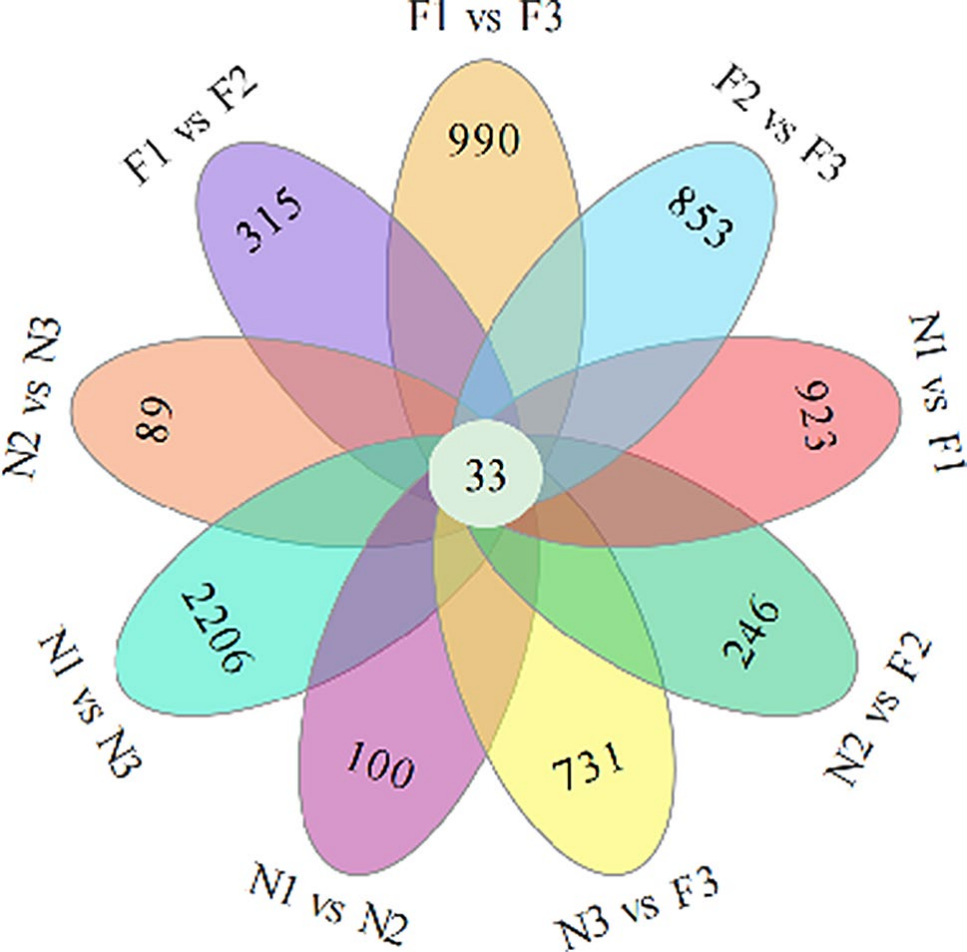
Venn diagram of DEGs in the nine comparison groups. The Venn diagram represents the overlapping DEGs among the nine comparison groups, and the number represents the unique number of DEGs in each comparison group

DEGs were identified using the criteria of |log2(FC)| ≥ 1 and FDR < 0.05, in nine pairwise comparisons: N1 vs. N2, N1 vs. N3, N2 vs. N3, F1 vs. F2, F1 vs. F3, F2 vs. F3, N1 vs. F1, N2 vs. F2, and N3 vs. F3. In the comparisons among N1, N2, and N3, N1 was designated as the control group. In the comparisons among F1, F2, and F3, F1 was designated as the control group. The total DEGs for each comparison were 4560, 12,563, 4546, 6060, 9479, 8432, 8475, 4661, and 7519, respectively. Among these, up-regulated genes numbered 2602, 7620, 1994, 2781, 5054, 5148, 3591, 2470, and 3480, while down-regulated genes totaled 1958, 4943, 2552, 3279, 4425, 3284, 4884, 2191, and 4039, respectively (Supplementary Fig. 1). Gene overlap analysis revealed distinct sets of genes for each of the nine comparison groups, with 100, 2206, 89, 315, 990, 853, 923, 246, and 731 genes identified as unique to their respective groups (Fig. 3). 2206 DEGs were enriched in the comparison group N1 vs. N3, and these DEGs were included in 133 pathways. Among them, Metabolic pathways (ko01100, contains 1031 DEGs) and Biosynthesis of secondary metabolites (ko01110, contains 585 DEGs) have the most enriched DEGs. These two pathways are not involved in anthocyanin biosynthesis. Additionally, 33 DEGs were shared across all nine comparison groups. None of these genes was significantly associated with anthocyanin biosynthesis according to gene annotations, suggesting their involvement in other metabolic pathways in quinoa leaves.

### GO and KEGG enrichment analyses

GO enrichment analysis revealed the enrichment of GO terms in various comparisons. Specifically, in N1 vs. N2, N1 vs. N3, N2 vs. N3, F1 vs. F2, F1 vs. F3, N1 vs. F1, N2 vs. F2, and N3 vs. F3, the number of enriched DEGs in GO terms were 1489, 2107, 1421, 1660, 1857, 1797, 1815, 1436, and 1705, respectively. These enrichments encompassed BP with 1035, 1397, 962, 1148, 1251, 1249, 1235, 993, and 1136 DEGs; MF involving 324, 504, 333, 372, 431, 389, 410, 314, and 403 DEGs; and CC comprising 130, 206, 126, 140, 175, 159, 170, 129, and 166 DEGs (Supplementary Fig. 2).

**Fig. 4:**
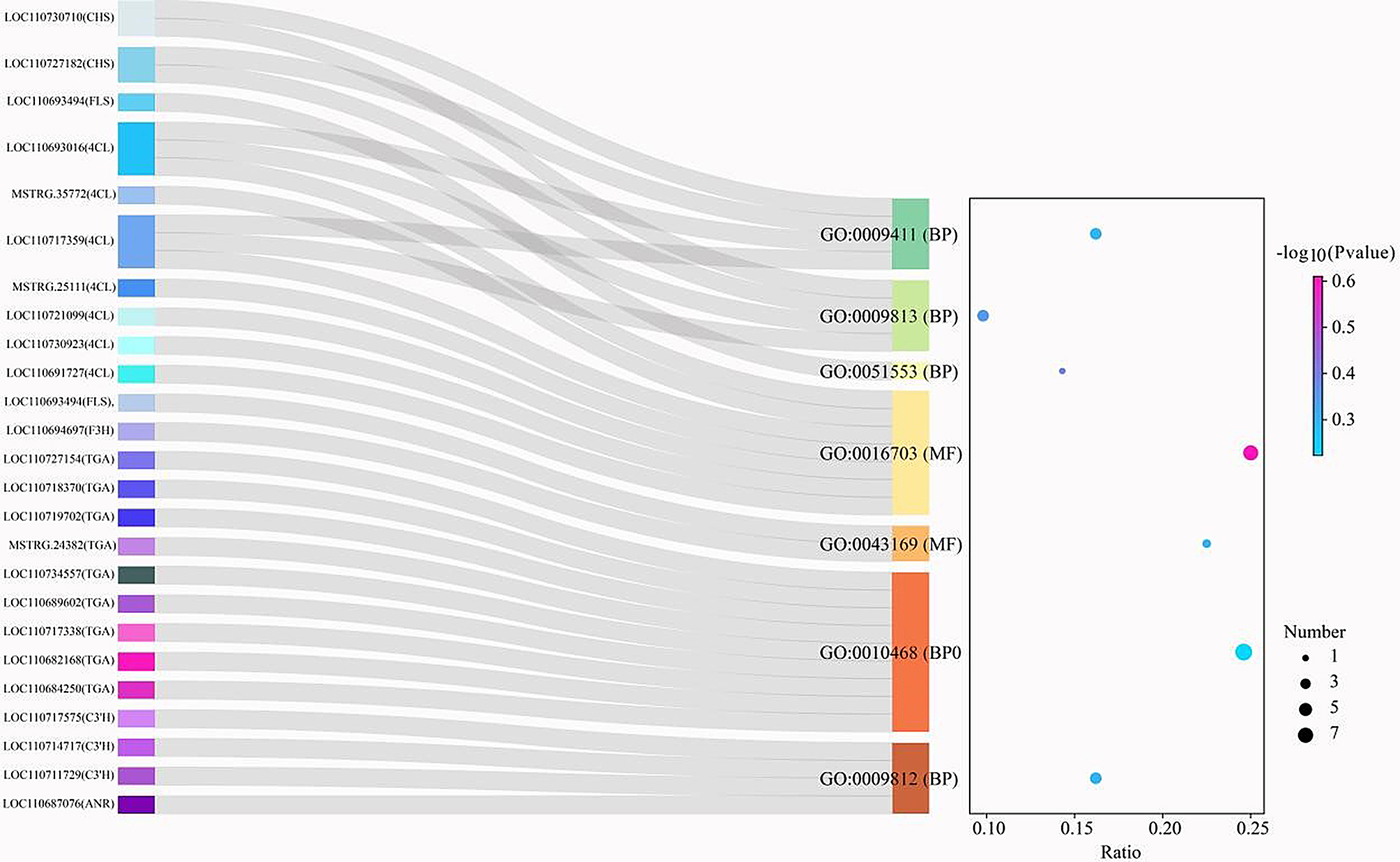
Sankey and dot plots of DEGs enriched in GO analysis of quinoa leaves. The Sankey map on the left delineates the genes associated with anthocyanin biosynthesis enriched in each GO team. The horizontal coordinate of the dot plot on the right is the ratio of each GO team, the vertical coordinate represents the -log10 (P_value), and the size of each circle represents the number of genes enriched in each GO team

Annotation analysis revealed that seven GO terms were closely associated with anthocyanin biosynthesis, primarily within BP and MF categories. For BP, the relevant GO terms included GO:0009812 (flavonoid metabolic process), GO:0010468 (regulation of gene expression), GO:0051553 (flavone biosynthetic process), GO:0009813 (flavonoid biosynthetic process), and GO:0009411 (response to UV). In the MF category, GO:0043169 (cation binding) and GO:0016703 (oxidoreductase activity) were identified. Furthermore, the differential expression of TFs *WRKY22*, *HY5*, and *TGA* was enriched in the GO:0010468 term (Fig. 4).

**Fig. 5:**
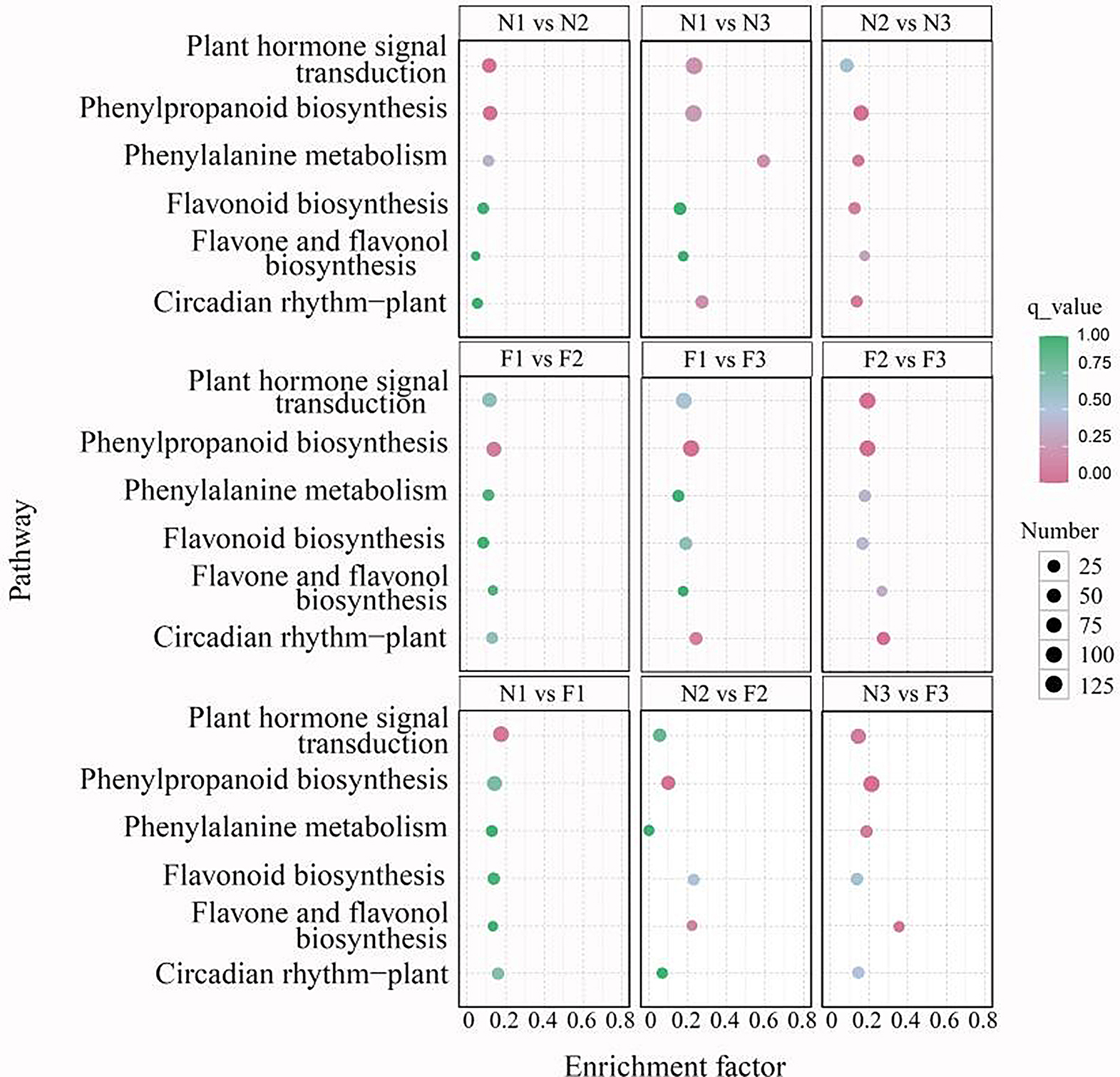
Bubble diagram of DEGs based on KEGG enrichment analysis. Note: The vertical axis represents the metabolic pathways obtained by KEGG enrichment analysis, and the horizontal axis represents enrichment factors. The six metabolic pathways (phenylalanine metabolism (ko00360), phenylpropanoid biosynthesis (ko00940), flavonoid biosynthesis (ko00941), plant hormone signal transduction (ko04075), flavone and flavonol biosynthesis (ko00944), and circadian rhythm-plant (ko04712)) are significantly associated with anthocyanin biosynthesis. Source: www.kegg.jp/kegg/kegg1.html

KEGG enrichment analyses in various comparisons (N1 vs. N2, N1 vs. N3, N2 vs. N3, F1 vs. F2, F1 vs. F3, N1 vs. F1, N2 vs. F2 and N3 vs. F3) revealed the involvement of a total of 121, 133, 124, 125, 132, 131, 128, 124, and 131 significantly different metabolic pathways, respectively. Among these, six pathways were significantly associated with anthocyanin biosynthesis: phenylalanine metabolism (ko00360), phenylpropanoid biosynthesis (ko00940), flavonoid biosynthesis (ko00941), plant hormone signal transduction (ko04075), flavone and flavonol biosynthesis (ko00944), and circadian rhythm-plant (ko04712). Phenylpropanoid biosynthesis exhibited the highest gene enrichment in all comparisons, with 62, 120, 83, 72, 114, 100, 74, 52, and 111 genes, respectively. The highest enrichment factors for flavonoid biosynthesis were 0.056, 0.222, 0.222, 0.167, 0.222, 0.333, 0.167, 0.278, and 0.444, respectively (Fig. 5).

The structural genes identified in the nine comparison groups included *4CL*, caffeoyl-CoA O-methyltransferase (*C-COA*), *HCT*, *CHS*, *CHI*, anthocyanidin reductase (*ANR*), Flavonoid 3’ monooxygenase (*CYP75B1*), anthocyanidin 3-O-glucoside-2-“-O-xylosyltransferase (*UGT79B1*), flavonol-3-O-glucoside (*FG3*), flavonol-3-O-glucoside L-rhamnosyltransferase (*FG2*), and trans-cinnamate 4-monooxygenase (*CYP73A*). Furthermore, TFs screened through KEGG enrichment included *MYC2* and *bHLH14* of the bHLH family, and *HY5* and *TGA* of the bZIP family, which were enriched in plant hormone signal transduction (ko04075), circadian rhythm-plant (ko04712), and MAPK signaling pathway-plant (ko04016).

### Metabolomic analysis

**Fig. 6:**
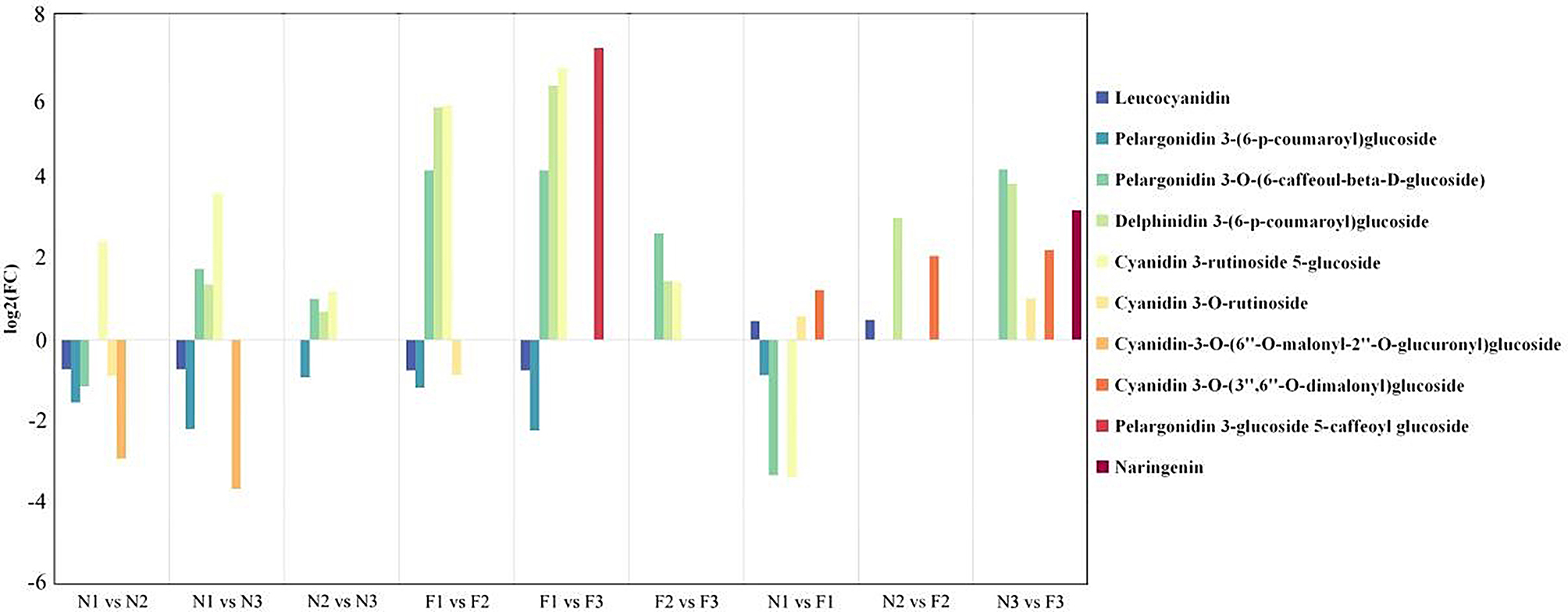
Expression regulation of DAMs in different comparison groups. This legend presents the number of DAMs, their difference multiples (log2FC), and the name of the key DAMs in the nine comparison groups. In the comparisons among N1, N2, and N3, N1 was designated as the control group. In the comparisons among F1, F2, and F3, F1 was designated as the control group

Metabolomic analysis of the sample revealed the detection of 1886, 2354, 1281, 2230, 2621, 2405, 1712, 1553 and 1878 metabolites in N1 vs. N2, N1 vs. N3, N2 vs. N3, F1 vs. F2, F1 vs. F3, F2 vs. F3, N1 vs. F1, N2 vs. F2 and N3 vs. F3 comparison groups. Among these metabolites, there were 381, 741, 808, 940, 1149, 1254, 837, 1054 and 1175 up-regulated; 1505,1613 ,473, 1290, 1472, 1151, 875, 499 and 703 down-regulated in each comparison groups, respectively (Supplementary Table 3). Statistical analysis of all metabolite enriched pathways showed that most metabolite pathways were enriched into metabolism components. (Supplementary Fig. 3). The screening criteria used for identifying these DAMs included VIP > 1, |log2(FC)| ≥ 1 and *P* < 0.05. The results showed that 97, 100, 86, 99, 106, 101, 101, 95 and 96 pathways of DAMs were enriched in 9 comparison groups, respectively. The pathway associated with anthocyanin biosynthesis is the anthocyanin biosynthesis pathway (ko00942). Metabolomics analysis of ko00942 pathway indicated that there were 6, 6, 4, 6, 6, 3, 6, 3, and 5 DAMs in N1 vs. N2, N1 vs. N3, N2 vs. N3, F1 vs. F2, F1 vs. F3, F2 vs. F3, N1 vs. F1, N2 vs. F2, and N3 vs. F3, respectively. Notably, cyanidin, delphinidin, pelargonidin, and their derivatives were identified in DAMs (Fig. 6). KEGG enrichment analysis indicated that the anthocyanin biosynthesis pathway (ko00942) was enriched. Analysis of log2(FC) values highlighted pelargonidin 3-O-(6-caffeoyl-beta-D-glucoside), delphinidin 3-(6-p-coumaroyl) glucoside, and cyanidin 3-rutinoside 5-glucoside as highly accumulated in M146, indicating their crucial role in anthocyanin biosynthesis and the subsequent increase in total anthocyanin content. The concentration of cyanidin 3-O-(3*’’*,6*’’*-O-dimalonyl) glucoside exhibited a more consistent increase in M146 than in M202 during the same period. This suggested that cyanidin 3-glucoside plays a pivotal role as a key metabolite in the generation of pink leaves. Additionally, cyanidin 3-O-rutinoside was up-regulated during F1 and F3, and leucocyanidin was up-regulated during F1 and F2, implying its involvement in pink quinoa leaf formation. Naringenin displayed initial differential expression in the N3 and F3 stages, suggesting its significance in later stages of pink quinoa leaf development. The analysis of metabolite content between M146 and M202 revealed higher levels of both anthocyanin category and content in pink quinoa leaves than in emerald green quinoa leaves. This suggests that quinoa tea products derived from pink quinoa leaves in the F3 period exhibited the highest anthocyanin content with superior nutritional value.

### Integrated transcriptomic and metabolomic analyses

**Fig. 7:**
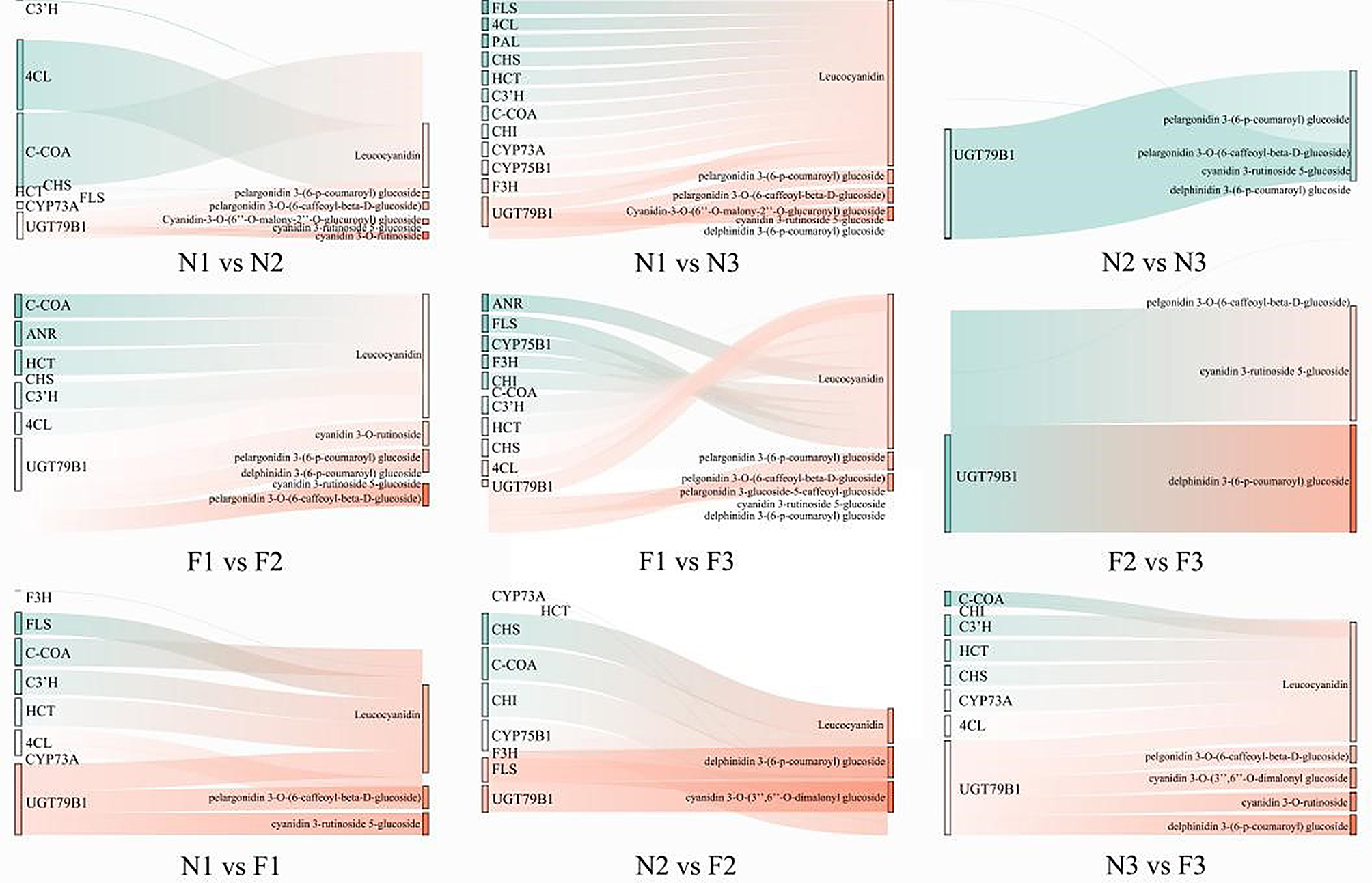
Sankey diagram. The left side represents the DEGs associated with the DAMs. The right side represents the DAMs associated with the DEGs

Integrated transcriptomic and metabolomic analysis revealed significant correlations between DEGs and DAMs within the phenylpropanoid biosynthesis (ko00940), flavonoid biosynthesis pathway (ko00941), and anthocyanin biosynthesis pathway (ko00942) (Fig. 7). The correlation network plot illustrated eight positive correlations (*4CL*, *C-COA*, *CYP73A*, and *UGT79B1* positively correlated with Leucocyanidin; *UGT79B1* positively correlated with pelargonidin 3-(6-p-coumaroyl) glucoside, pelargonidin 3-O-(6-caffeoyl-beta-D-glucoside), cyanidin-3-O-(6*’’*-O-malonyl-2*’’*-O-glucuronyl) glucoside, and cyanidin 3-O-rutinoside) and five negative correlations (*CHS*, *HCT*, *FLS*, and *C3’H* negatively correlated with leucocyanidin; *UGT79B1* negatively correlated with cyanidin 3-rutinoside 5-glucoside) within the N1 vs. N2 comparison group. The correlation network plot illustrated fifteen positive correlations (*4CL*, *FLS*, *PAL*, *CHS*, *HCT*, *C3’H*, *C-COA*, *CHI*, *CYP73A*, *CYP75B1*, *UGT79B1* and *F3H* positively correlated with Leucocyanidin; *UGT79B1* positively correlated with Pelargonidin 3-(6-p-coumaroyl) glucoside, Pelargonidin 3-O-(6-caffeoyl-beta-D-glucoside) and Cyanidin-3-O-(6*’’*-O-malonyl-2*’’*-O-glucuronyl) glucoside) and two negative correlations (*UGT79B1* negatively correlated with Cyanidin 3-rutinoside 5-glucoside and Delphinidin 3-(6-p-coumaroyl) glucosid) within the N1 vs. N3 comparison group. The correlation network plot illustrated one positive correlations (*UGT79B1* positively correlated with Pelargonidin 3-(6-p-coumaroyl) glucoside) and three negative correlations (*UGT79B1* negatively correlated with Pelargonidin 3-O-(6-caffeoyl-beta-D-glucoside), Cyanidin 3-rutinoside 5-glucoside and Delphinidin 3-(6-p-coumaroyl) glucoside)) with in the N2 vs. N3 comparison group. The correlation network plot illustrated eight positive correlations (*C-COA*, *HCT*, *C3’H*, *4CL*, and *UGT79B1* positively correlated with leucocyanidin; *UGT79B1* positively correlated with Cyanidin 3-O-rutinoside, Pelargonidin 3-(6-p-coumaroyl) glucoside and Pelargonidin 3-O-(6-caffeoyl-beta-D-glucoside)) and four negative correlations (*ANR* and *CHS* negatively correlated with leucocyanidin; *UGT79B1* negatively correlated with Delphinidin 3-(6-p-coumaroyl) glucoside and Cyanidin 3-rutinoside 5-glucoside ) with in the F1 vs. F2 comparison group. The correlation network plot illustrated twelve positive correlations (*ANR*, *FLS*, *CYP75B1*, *F3H*, *CHI*, *C3’H*, *HCT*, *CHS*, *4CL* and *UGT79B1* positively correlated with leucocyanidin; *UGT79B1* positively correlated with Pelargonidin 3-(6-p-coumaroyl) glucoside and Pelargonidin 3-O-(6-caffeoyl-beta-D-glucoside)) and four negative correlations (*C-COA* negatively correlated with leucocyanidin; *UGT79B1* negatively correlated with Delphinidin 3-(6-p-coumaroyl) glucoside, Cyanidin 3-rutinoside 5-glucoside and Pelargonidin 3-glucoside 5-caffeoyl glucoside ) with in the F1 vs. F3 comparison group. The correlation network plot illustrated two positive correlations (*UGT79B1* positively correlated with Delphinidin 3-(6-p-coumaroyl) glucoside and Cyanidin 3-rutinoside 5-glucoside) and one negative correlations (*UGT79B1* negatively correlated with Pelargonidin 3-O-(6-caffeoyl-beta-D-glucoside) ) with in the F2 vs. F3 comparison group. The correlation network plot illustrated eight positive correlations (*FLS*, *C-COA*, *C3’H*, *HCT*, *4CL* and *UGT79B1* positively correlated with leucocyanidin; *UGT79B1* positively correlated with Pelargonidin 3-O-(6-caffeoyl-beta-D-glucoside) and Cyanidin 3-rutinoside 5-glucoside) and two negative correlations (*F3H* and *CYP73A* negatively correlated with leucocyanidin) with in the N1 vs. F1 comparison group. The correlation network plot illustrated five positive correlations (*CHS*, *C-COA*, *CHI*, *CYP75B1* and *FLS* positively correlated with leucocyanidin) and six negative correlations (*HCT*, *CYP73A*, *F3H* and *UGT79B1* negatively correlated with leucocyanidin; *UGT79B1* negatively correlated with Delphinidin 3-(6-p-coumaroyl) glucoside and Cyanidin 3-O-(3*’’*,6*’’*-O-dimalonyl) glucoside) with in the N2 vs. F2 comparison group. The correlation network plot illustrated eleven positive correlations (*C-COA*, *C3’H*, *HCT*, *CHS*, *CYP73A*, *4CL* and *UGT79B1* positively correlated with leucocyanidin; *UGT79B1* positively correlated with Pelargonidin 3-O-(6-caffeoyl-beta-D-glucoside), Cyanidin 3-O-(3*’’*,6*’’*-O-dimalonyl) glucoside, Cyanidin 3-O-rutinoside and Delphinidin 3-(6-p-coumaroyl) glucoside) and one negative correlations (*CHI* negatively correlated with leucocyanidin) with in the N3 vs. F3 comparison group.

### Analysis of transcription factors

In the TF analysis, 2645 DEGs spanning 55 TF families were identified. Notably, the bHLH, bZIP, and WRKY TF families, which are involved in anthocyanin regulation, were highlighted. Specifically, the bHLH and bZIP TF families exhibited significant enrichment of 201 and 91 DEGs, respectively. The WRKY TF family was enriched in 100 DEGs (Supplementary Fig. 4). Key TFs included *MYC2* and *bHLH14* of the bHLH family, *HY5* and *TGA* of the bZIP family, and *WRKY24* and *WRKY46* of the WRKY family. These TFs were found to be expressed to varying degrees during quinoa leaf growth, and they were associated with four metabolic pathways: circadian rhythm-plant (ko04712), plant hormone signal transduction (ko04075), MAPK signaling pathway-plant (ko04016), and plant-pathogen interaction (ko04626) (Supplementary Table 4).

### qRT-PCR verification of DEGs

**Fig. 8:**
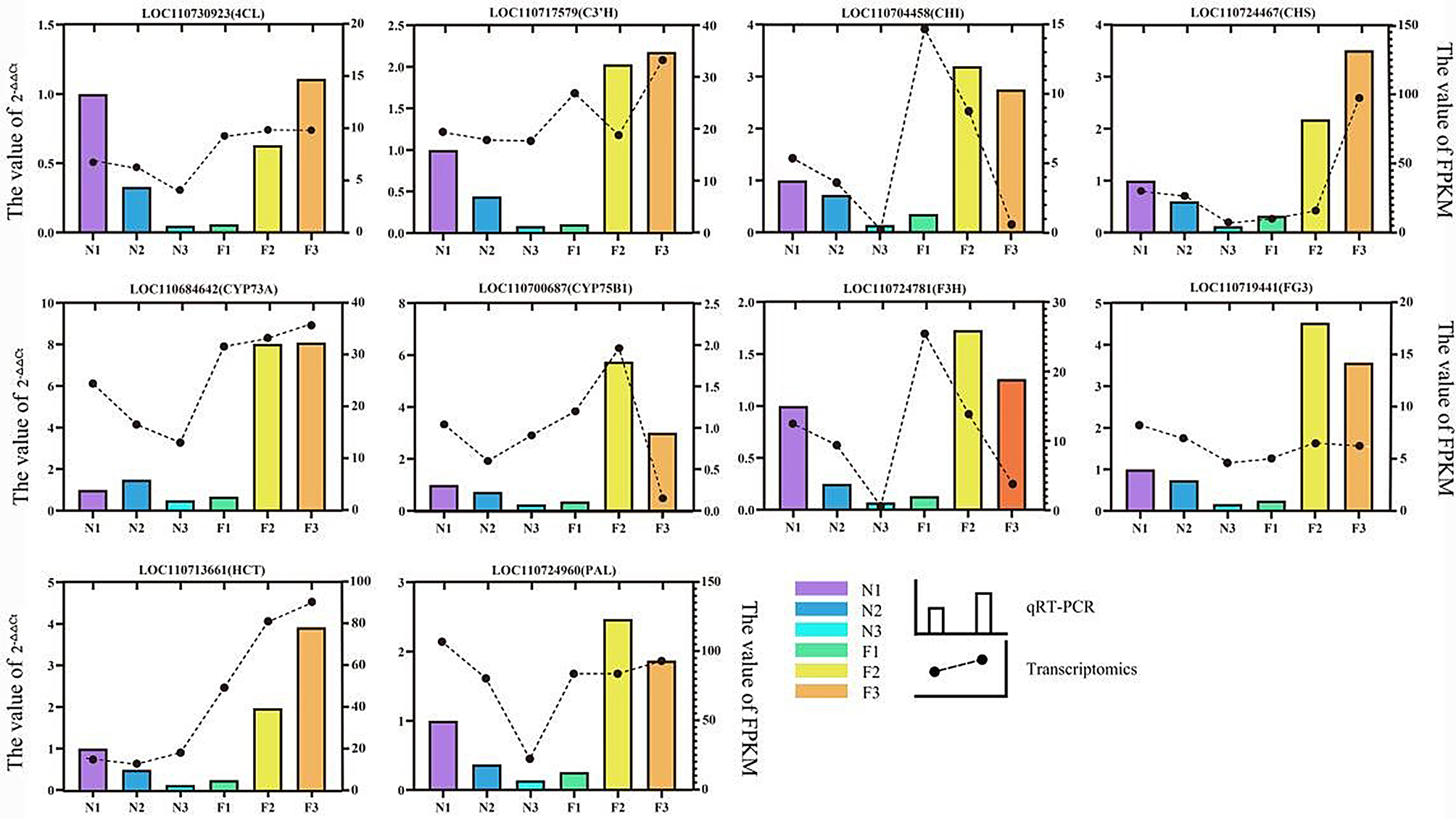
qRT-PCR validation of 10 key genes. The left Y-axis represents the relative expression of samples in qRT-PCR, and the right Y-axis represents the FPKM value in transcriptomics. The column represents the relative qRT-PCR expression of each component in each comparison group, corresponding to the left Y-axis. The black circle represents the FPKM value of each component in each comparison group, corresponding to the right Y-axis

To assess the authenticity and reliability of the transcriptome data and the extent of DEGs. Ten DEGs across the six enriched pathways were selected for validation via qRT-PCR, including *4CL* (LOC110730923), *C3’H* (LOC110717575), *CHI* (LOC110704458), *CHS* (110,724,467), *CYP75B1* (LOC110700687), *F3H* (LOC110724781), *FG3* (LOC110719441), *HCT* (LOC110713661), *PAL* (LOC110724960) and *CYP73A* (LOC110684642). The qRT-PCR analysis revealed a successive linear down-regulation trend in the expression of *4CL*, *C3’H*, *CHI*, *CHS*, *F3H*, *FG3*, and *PAL* from N1 to N3 in green leaves. This pattern was consistent with the findings obtained from transcriptomic analysis. The qRT-PCR analysis revealed a successive up-down regulation of *4CL*, *CHS*, *CYP73A*, *FG3*, and *HCT* genes from F1 to F3 in pink leaves, which corroborated with the transcriptomics results. The qRT-PCR trends of *CYP73A* in the N2, *CYP75B1* and *HCT* in the N3, *CHI* and *F3H* in the F1 and *C3’H* and *PAL* in the F2 were inconsistent with transcriptomics results. From F1 to F3, *C3’H* exhibited a u-shaped trend, and *CHI* showed a linear downward trend in the transcriptomics data. However, in the qRT-PCR results, *C3’H* displayed a linear upward trend, and *CHI* exhibited an inverted “V” trend. From N1 to N3, *CYP73A* showed down-regulated trend and *CYP75B1* showed a V-shaped trend in transcriptomics data. However, in the qRT-PCR results, *CYP73A* displayed a V-shaped trend, and *CYP75B1* showed a down-regulated trend (Fig. 8). In summary, the qRT-PCR verification results achieved the expected purpose of the experiment, although the expression of certain candidate genes deviated from the transcriptomic results at specific developmental stages. In addition to the individual structural genes controlling single physiological responses, gene expression patterns also involve gene interactions, pleiotropism, and multigenetic effects. In addition, gene expression is influenced by various factors such as the content of metabolic initiators, substrate concentration and TFs.

## Discussion

### Phenotypic analysis of quinoa leaves

Phenotypic observations and analysis of anthocyanin content revealed higher levels in pink quinoa leaves than in emerald green leaves across all growth stages. This trend was consistent with findings in ornamental kale [35], purple and green perilla leaves [36], and quinoa grains [37], indicating a close relationship between the phenotype and anthocyanin content. Color difference analysis showed that ΔE values between adjacent periods were all below 4, indicating imperceptible color changes to the naked eye. However, at 70 DAG (N1, F1) and 110 DAG (N3, F3), the ΔE values exceeded 6, indicating noticeable color changes. Previous studies have demonstrated the accumulation of anthocyanins during senescence [38]. According to the anthocyanin content measurements in this study, as quinoa leaves matured after discoloration, there was a progressive increase in anthocyanin content, resulting in a slight deepening of color at 110 DAG. This trend in anthocyanin content variation was consistent with the observed color difference.

### Analysis of key DEGs

*4CL* is a pivotal regulator upstream of the flavonoid metabolic pathway, which regulates anthocyanin and flavonoid synthesis. *4CL* exhibits notably varied substrate preferences and specificities [39]. *CHI* is the second key enzyme in flavonoid synthesis, and its up-regulation can increase the synthesis of flavonoid secondary metabolites in plants and catalyze intramolecular cyclization [40]. Zhang et al. [41] demonstrated a positive correlation between the expression of *4CL* and the formation of yellow peel coloration of melon. Ho et al. [42] demonstrated that low expression of *CHI* and *F3H* can promote hyperpigmentation. In this study, *4CL* was significantly down-regulated in the N3 period and significantly up-regulated in the F3 period, which indicating that the high expression of *4CL* might promote the formation of pink leaves of quinoa, while the low expression might maintain the green leaves of quinoa. This phenomenon indicates that *4CL* has substrate preference, which was consistent with the result of Zhang et al.‘s study [41]. Conversely, *CHI* expression displayed a consistent reduction across all periods, which can promote hyperpigmentation. *CHI* positively correlating with *bHLH14*. We speculated that *bHLH14* regulates *CHI* during the discoloration phase and affect the color change of quinoa leaves. The precise regulatory mechanisms require further investigation.

Fukusaki et al. [43] employed RNAi to suppress *CHS* in *Torenia lybrida*, resulting in transgenic plants with white and grey flowers. Similar successes have been documented in the leaves of *Gerbera hybrida* [44], *Petunia* [45], and *Gentian corolla* [46]. These results consistently indicated a positive correlation between *CHS* expression and vividness of plant color. In this study, *CHS* expression of pink quinoa leaves was significantly higher than emerald green leaves. This consistent with the results of previous studies. Hinderer et al. [47] demonstrated that *CHS* activity in carrots was inhibited by naringin and chalcone, influenced by downstream flavonoids in the metabolic pathway. In this study, the N3 vs. F3 comparison group displayed notable differential naringin expression for the first time. Moreover, *CHS* expression in the N3 and F3 periods was significantly lower than that in the N1, N2, F1 and F2 periods, implying that the increased naringin expression in quinoa leaves also exerted inhibitory effects on *CHS* activity.

*PAL* predominantly facilitates the catalysis of trans-cinnamic acid production from L-phenylalanine ammonia, serving as the pivotal entry- and rate-limiting enzyme within the phenylpropane pathway in plants [48]. Xue et al. [49] reported the up-regulated expression of *PAL* can promote the pink testa in peanut. Mattus-Araya et al. [50] reported high expression of *PAL* can speeds up the progress of color in developing *F. chiloensis* Fruit. In this study, *PAL* expression was significantly down-regulated during the N2 and N3 stages, with expression levels notably higher in the F3 stage than in N3 and s in pink quinoa leaves. This indicates that *PAL* may play a role in maintaining the persistent pink hue observed in quinoa leaves, whereas its down-regulated may contribute to the maintenance of green coloration.

*F3H* plays a pivotal role in catalyzing the hydroxylation of flavonoids, which is a crucial step in anthocyanin biosynthesis [51]. Studies on alfalfa and carnations [52, 53] have indicated that reduced *F3H* expression can lead to deeper coloration. In this study, both transcriptomic analysis and qRT-PCR validation consistently demonstrated a gradual decrease in *F3H* expression across distinct leaf growth periods within the same quinoa variety. This trend was consistent with the results of chromatic value in quinoa leaves. The results of this study are consistent with those of previous studies in this field and indicated that the low expression of *F3H* could promote the deepening of leaves color. However, the expression level of *F3H* in pink quinoa leaves was significantly higher than that in green quinoa leaves during the same period, suggesting that *F3H* and other genes jointly regulate the change of leaf color. The specific regulatory mechanism needs to be further study.

*HCT* has been identified as a key regulator of anthocyanin biosynthesis in quinoa leaves. *HCT* facilitates the conversion of 4-coumaroyl CoA into caffeoyl-CoA. In addition, Liu [35] elucidated the branching pathway of anthocyanin biosynthesis by inducing concurrent alterations in anthocyanin, chlorophyll, and carotenoid contents, resulting in the emergence of green spots within ornamental kale exhibiting pink leaves. In this way, *HCT* transforms 4-coumaroyl CoA into caffeoyl-CoA, which subsequently undergoes synthesis through *CHS*, *F3H*, *DFR*, and anthocyanidin synthase (*ANS*) to yield anthocyanins. This established anthocyanin synthesis pathway is consistent with our current findings. Studies have indicated that silencing *HCT* not only suppresses lignin biosynthesis but also leads to activation of *CHS*, resulting in the accumulation of various flavonol glycosides and acylated anthocyanins [54]. Li et al. [55] showed that *HCT* exhibited substrate preference. Transcriptomic analysis in this study revealed continuous expression of *HCT* in emerald green leaves, with another up-regulation observed during the F3 period. This suggests that elevated *HCT* expression may facilitate the formation of pink leaves and promote substrate preference. Furthermore, in cultivar M146, there appears to be a propensity for caffeoyl-CoA formation, subsequently leading to the synthesis of various anthocyanins. However, in this study, no discernible relationship between *HCT* and *CHS* was observed, warranting further investigation to elucidate the specific regulatory mechanisms governing color change.

*FLS* catalyzes the conversion of dihydroflavonols to flavonols, a process that competes with *DFR* at a crucial branch point in the anthocyanin pathway. Ueyama et al. [56] demonstrated that up-regulation of *FLS* resulted in the production of white flowers in transgenic *Nierembergia* sp. In this study, significant down-regulation of *FLS* expression was observed in the F2 and F3 periods, with no notable difference detected in green quinoa leaves. This suggests that the down-regulated expression of *FLS* might be related to the formation of pink coloration in quinoa leaves.

### Analysis of key DAMs

Metabolites within ko00941 and ko00942 pathways affect quinoa leaf pigmentation. Naringenin, which is derived from naringenin chalcone through the action of *CHI*, is a crucial intermediary in anthocyanin synthesis. Motallebi et al. [57] demonstrated significance of naringin as a polyphenolic flavonoid compound with notable pharmacological effects in plants. This study revealed a significant differential expression of naringin during the N3 and F3 periods, suggesting its pivotal role in the transition from green to pink pigmentation. Furthermore, although no substantial alterations were observed in cyanidin 3-O-(3’’,6’’-O-dimalonyl) glucoside across different periods within the same variety, distinct variations were observed between different varieties during the same period. This indicated that its high expression may play a key role in the formation of pink pigmentation. Cyanidin-3-O-(6”-O-malonyl-2”-O-glucuronyl) glucoside was significantly down-regulated in all phases of green quinoa leaves, whereas no significant difference was observed in pink quinoa leaves and across different varieties during the same period. We propose that cyanidin-3-O-(6”-O-malonyl-2”-O-glucuronyl) glucoside serves as the key metabolite for maintaining green quinoa leaves. Yifan et al. [58] demonstrated a direct correlation between changes in blueberry surface color and anthocyanin concentration. In this study, we observed significant differences in the content of pelargonidin, delphinidin, and cyanidin derivatives within quinoa leaves. This suggests a potential association between the color transformation of quinoa leaves and the levels of these derivatives.

### Analysis of TFs

**Fig. 9:**
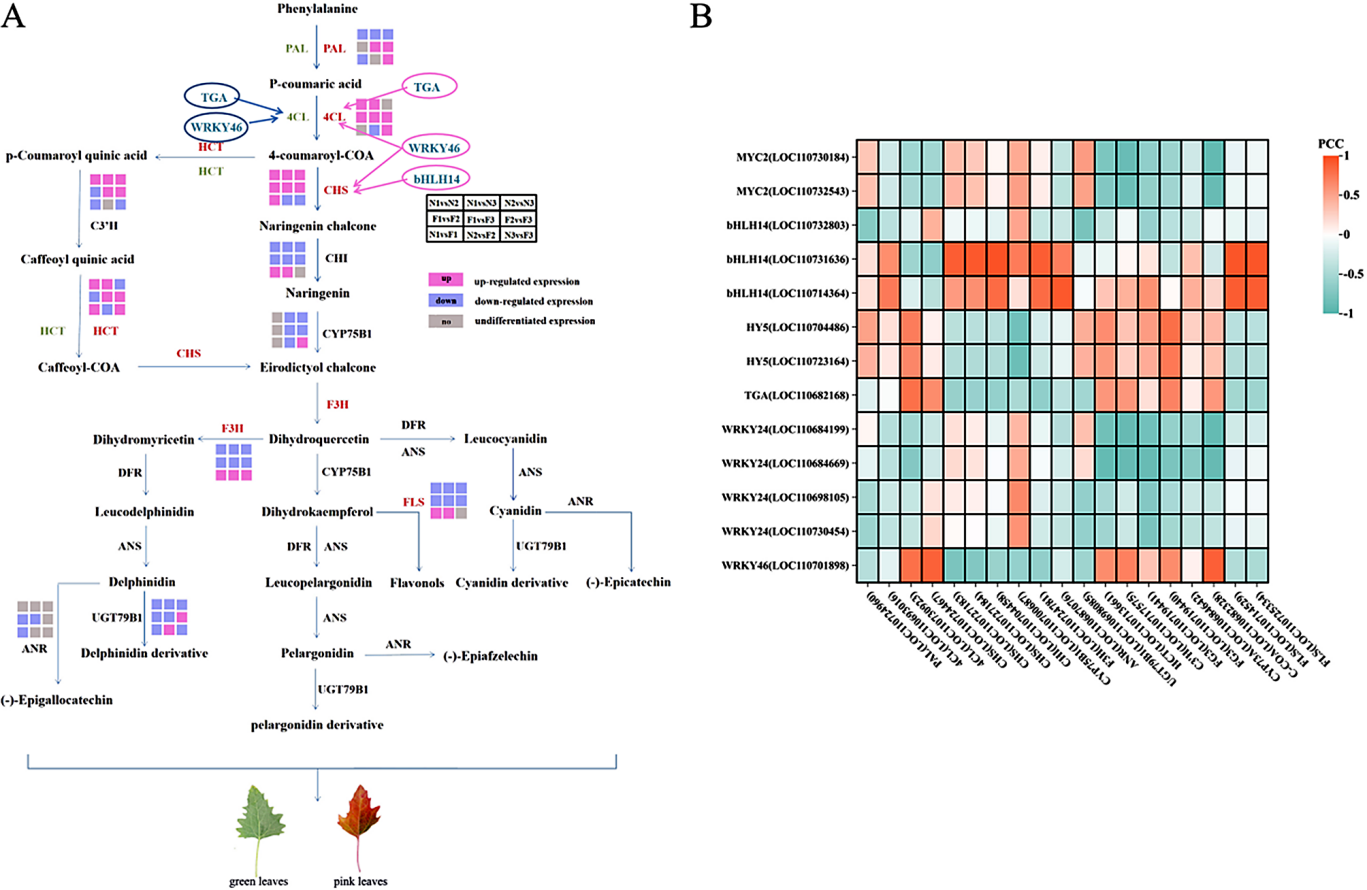
Anthocyanin biosynthesis pathway and correlation heat map between TF family genes and DEGs. Note: PAL is phenylalanine ammonia-lyase, CHS is chalcone synthase, 4CL is 4-coumarate CoA ligase, HCT is Hydroxycinnamoyltransferas, F3H is flavanone 3-hydroxylase, FLS is Flavonol synthase, CHI is chalcone isomerase, C-COA is caffeoyl-CoA O-methyltransferase, ANR is anthocyanidin reductase, CYP75B1 is Flavonoid 3’ monooxygenase, UGT79B1 is anthocyanidin 3-O-glucoside-2-“-O-xylosyltransferase, FG3 is flavonol-3-O-glucoside, ANS is anthocyanidin synthase, DFR is dihydroflavonol reductase, C3’H is 5-O-(4-coumaroyl)-D-quinate 3’-monooxygenase. (**A**): The anthocyanin biosynthetic pathway. Pink font indicates key genes closely associated with pink quinoa leaves. Green font indicates key genes closely associated with green quinoa leaves. The blue letters in the circle are TFs. (**B**): Heat map displaying Pearson’s correlation coefficients between TF genes and DEGs

In this study, six pivotal TFs related to anthocyanin biosynthesis were identified: *MYC2* and *bHLH14* from the bHLH family; *HY5* and *TGA* from the bZIP family; and *WRKY24* and *WRKY46* from the WRKY family. Based on transcriptomic-metabolomic data, an anthocyanin biosynthesis pathway specific to quinoa leaf coloration was elucidated (Fig. 9A). Pearson’s coefficient analysis was used to determine correlations between 13 differentially expressed TFs and 18 DEGs (Fig. 9B). The results showed a positive correlation between *bHLH14*, *WRKY46* and *CHS*. The high expression of *CHS* can enhance the expression of pink coloration in quinoa leaves. Therefore, *bHLH14* and *WRKY46* may work with *CHS* to positively regulate the formation of pink quinoa leaves and plays a crucial role in anthocyanin biosynthesis regulation. *TGA* and *WRKY46* exhibited a positive correlation with *4CL*, which showed *TGA* and *WRKY46* may regulate the formation of pink quinoa leaves or maintain the green color of quinoa leaves. The TF regulatory mechanisms are intricate, necessitating further in-depth investigation in subsequent studies. Additionally, a comprehensive exploration of the functional roles of the bHLH and WRKY families is warranted.

## Conclusions

In summary, fundamental insights into the anthocyanin synthesis pathways, related genes, and metabolites in the variously colored leaves of quinoa were acquired through integrated transcriptomics-metabolomics analyses. The primary findings of this study are outlined below: (1) Integrated transcriptomic and metabolomic analyses indicated that both flavonoid biosynthesis pathway (ko00941) and anthocyanin biosynthesis pathway (ko00942) were significantly associated with anthocyanin biosynthesis. (2) The diminished expression of *PAL*, *4CL*, and *HCT* decreased the formation of Cyanidin-3-O-(6”-O-malonyl-2”-O-glucuronyl) glucoside, leading to the emergence of emerald green leaves. Moreover, the lowered expression of *TGA* and *WRKY46* indirectly regulated *4CL* activity, serving as another important factor in maintaining the emerald green hue in leaves N1, N2, and N3. (3) Cyanidin 3-O-(3’’,6’’-O-dimalonyl) glucoside and naringenin are identified as the key metabolites responsible for the distinct coloration observed in the leaves of F1, F2, and F3. The high expression of *PAL*, *CHS*, *4CL* and *HCT*, as well as the low expression of *F3H* and *FLS* may be the key causes in the formation of pink leaves. In addition, *bHLH14*, *WRKY46*, and *TGA* indirectly affected the activities of *CHS* and *4CL*, collectively regulating the levels of cyanidin 3-O-(3*’’*, 6*’’*-O-dimalonyl) glucoside and naringenin.

### Electronic supplementary material

Below is the link to the electronic supplementary material.

Supplementary Material 1

Supplementary Material 2

Supplementary Material 3

Supplementary Material 4

Supplementary Material 5

Supplementary Material 6

Supplementary Material 7

Supplementary Material 8

## Data Availability

The datasets presented in this study can be found in online repositories. The names of the repository/repositories and accession number(s) can be found below: https://www.ncbi.nlm.nih.gov/, PRJNA986269.

## Abbreviations

DEGs: differentially expressed genes
DAMs: differentially accumulated metabolites
TFs: transcription factors
MYB: v-myb avian myeloblastosis viral oncogene homolog
bHLH: basic helix-loop-helix
70 DAG: 70 days after germination
90 DAG: 90 days after germination
110 DAG: 110 days after germination
KEGG: Kyoto encyclopedia of genes and genomes
GO: Gene ontology
FPKM: Fragments Per Kilobase per Million
FC: Fold change
PCR: Polymerase chain reaction
qRT-PCR: Real-time quantitative PCR
CDS: Code sequence
FDR: False discovery rate
