# Supplementary material for: Integrated transcriptomic and metabolomic analyses reveals anthocyanin biosynthesis in leaf coloration of quinoa (Chenopodium quinoa Willd.)
Source: BMC Plant Biol. 2024 Mar 20;24:203. doi: 10.1186/s12870-024-04821-2 (PMC10953167; doi:10.1186/s12870-024-04821-2)
Supplement: Supplementary file 1 — Supplementary Material 1 [file 12870_2024_4821_MOESM1_ESM.docx]

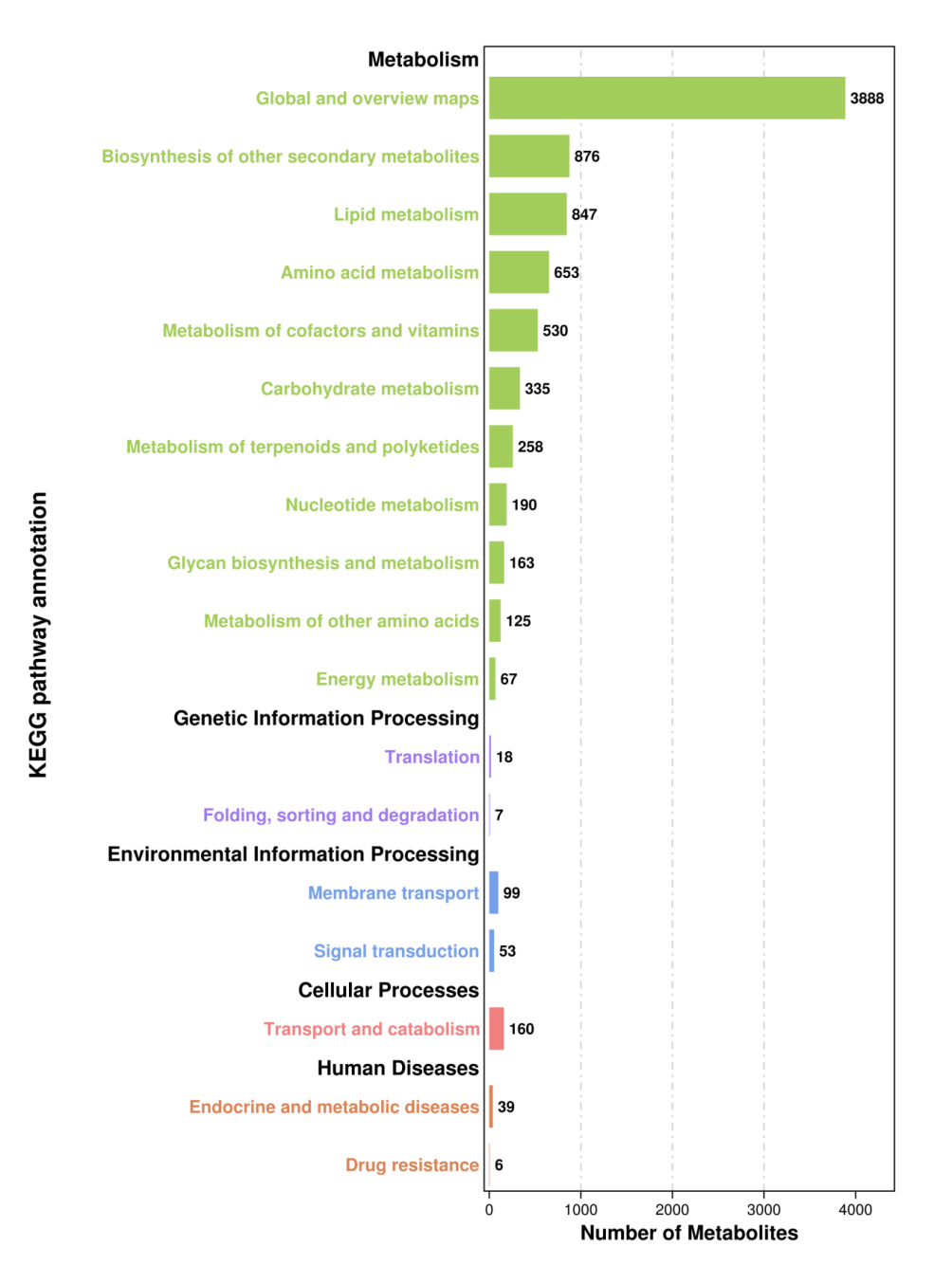


Supplementary Figure 3. Histogram of number metabolite enrichment of KEGG pathway. The horizontal coordinate is the number of metabolites enriched by each component. The ordinate is enriched to KEGG pathways annotation.
