# Supplementary material for: Integrated transcriptomic and metabolomic analyses reveals anthocyanin biosynthesis in leaf coloration of quinoa (Chenopodium quinoa Willd.)
Source: BMC Plant Biol. 2024 Mar 20;24:203. doi: 10.1186/s12870-024-04821-2 (PMC10953167; doi:10.1186/s12870-024-04821-2)
Supplement: Supplementary file 2 — Supplementary Material 2 [file 12870_2024_4821_MOESM2_ESM.docx]

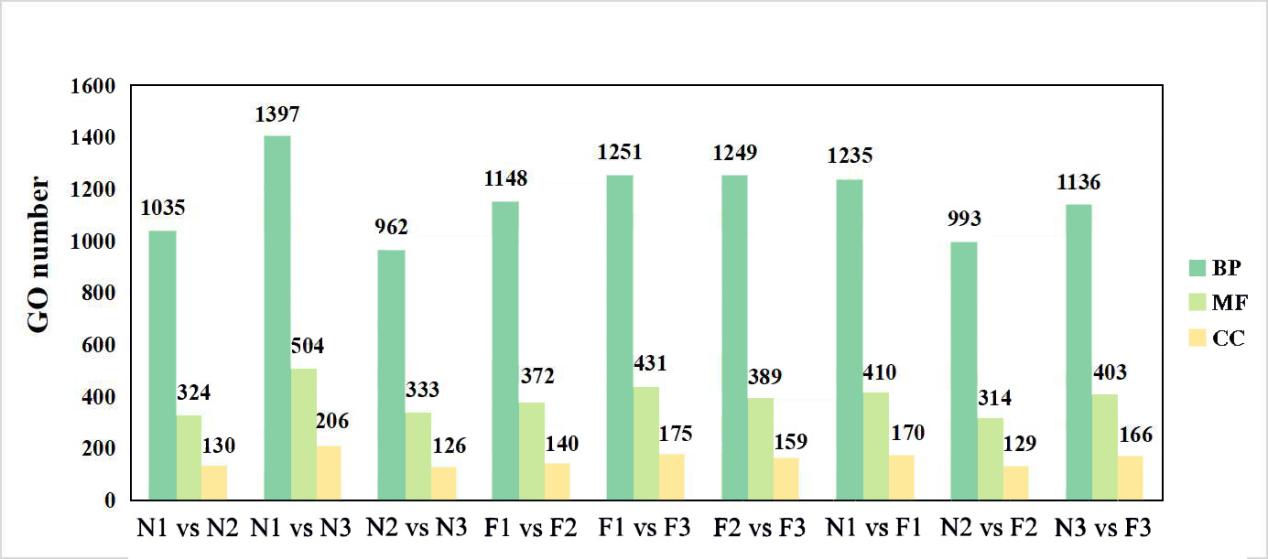


Supplementary Figure 2. Statistical diagram of GO terms enriched for each comparison group. Note: The vertical axis represents the number of GO terms enriched in each comparison group, and the horizontal axis represents each comparison group. The yellow, green, and blue columns represent the BP, MF, and CC components, respectively.
