# Supplementary material for: Integrated transcriptomic and metabolomic analyses reveals anthocyanin biosynthesis in leaf coloration of quinoa (Chenopodium quinoa Willd.)
Source: BMC Plant Biol. 2024 Mar 20;24:203. doi: 10.1186/s12870-024-04821-2 (PMC10953167; doi:10.1186/s12870-024-04821-2)
Supplement: Supplementary file 3 — Supplementary Material 3 [file 12870_2024_4821_MOESM3_ESM.docx]

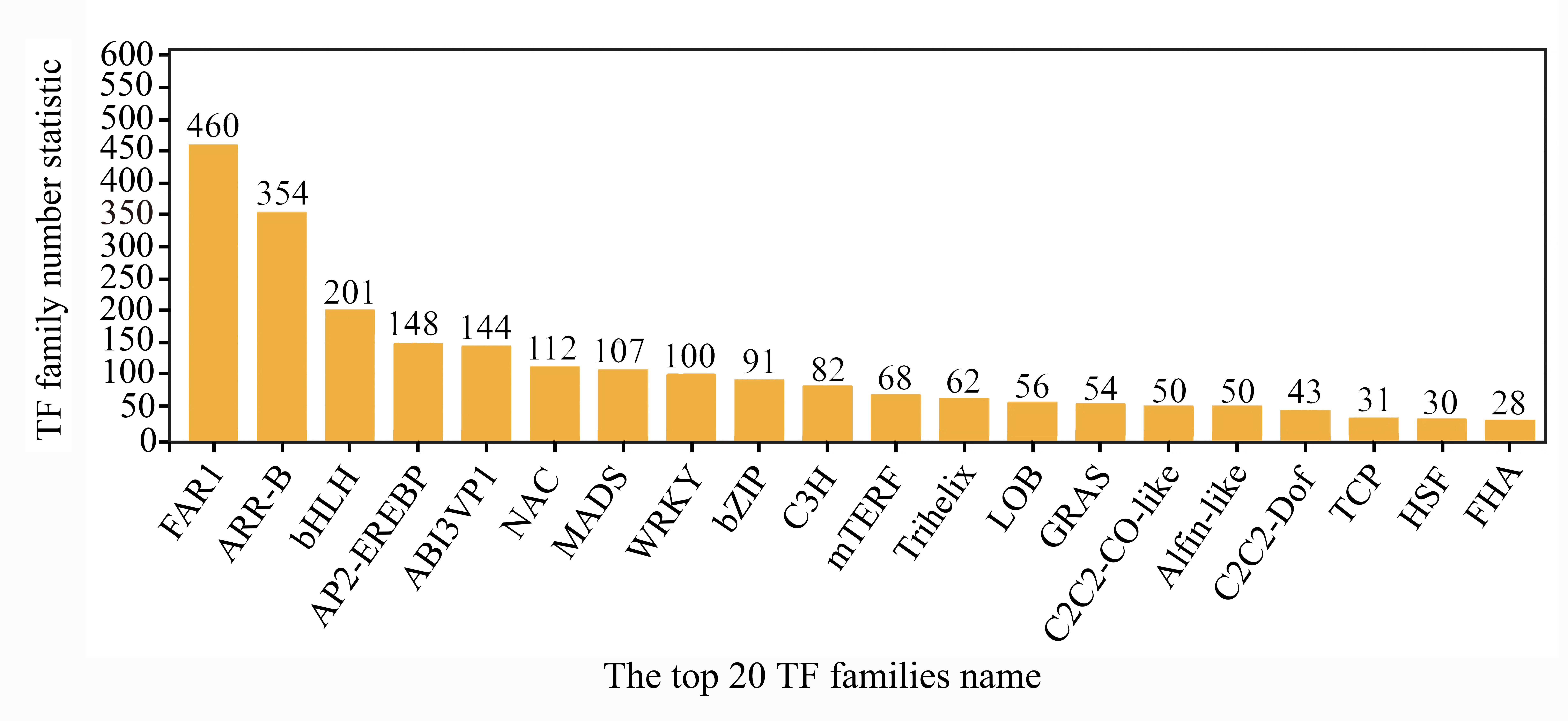


Supplementary Figure 4. Number of the top 20 TF families. The top 20 TF families with the highest numbers of differential genes were enriched. The horizontal coordinates indicate the names of the TF families, and the vertical coordinates indicate the number of differential genes enriched by each TF family.
