# Supplementary material for: Integrated transcriptomic and metabolomic analyses reveals anthocyanin biosynthesis in leaf coloration of quinoa (Chenopodium quinoa Willd.)
Source: BMC Plant Biol. 2024 Mar 20;24:203. doi: 10.1186/s12870-024-04821-2 (PMC10953167; doi:10.1186/s12870-024-04821-2)
Supplement: Supplementary file 4 — Supplementary Material 4 [file 12870_2024_4821_MOESM4_ESM.docx]

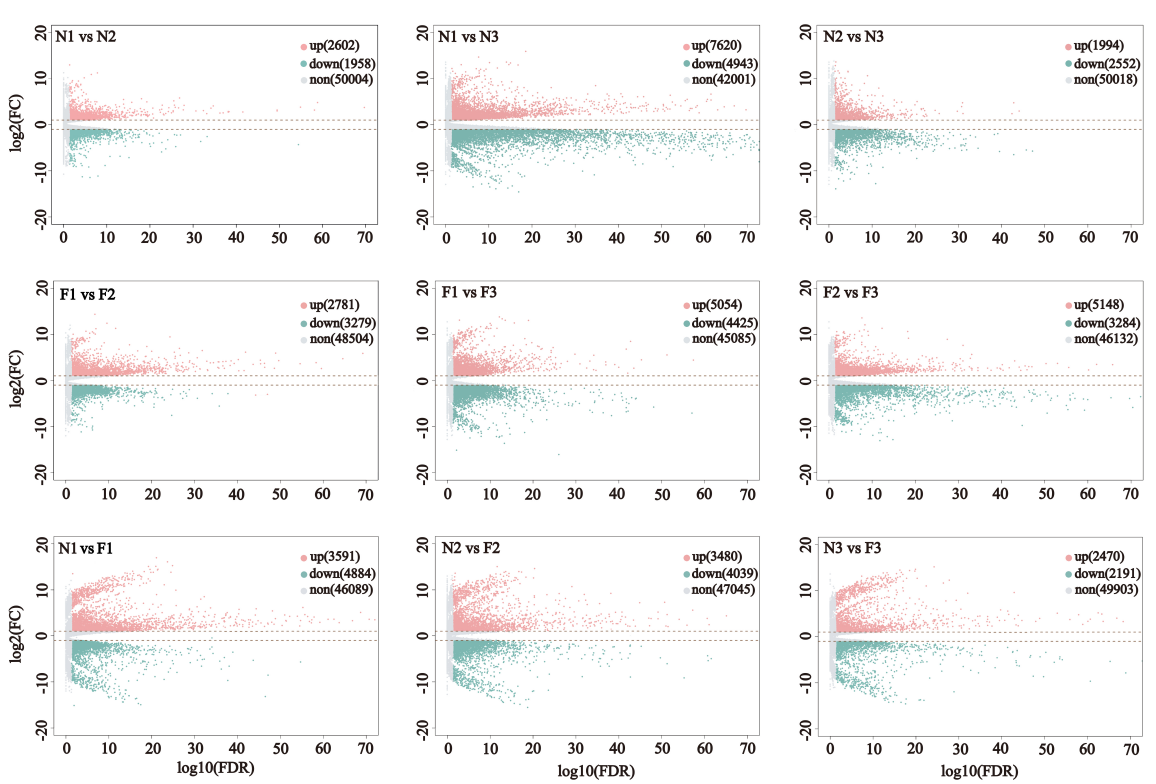


Supplementary Figure 1. M-versus-A plot (MA) diagrams of DEGs in the nine comparison groups. ‘Up’ represents up-regulated expression, ‘down’ represents down-regulated expression, and ‘non’ represents indifferent genes.
