# Supplementary material for: Integrated transcriptomic and metabolomic analyses reveals anthocyanin biosynthesis in leaf coloration of quinoa (Chenopodium quinoa Willd.)
Source: BMC Plant Biol. 2024 Mar 20;24:203. doi: 10.1186/s12870-024-04821-2 (PMC10953167; doi:10.1186/s12870-024-04821-2)
Supplement: Supplementary file 5 — Supplementary Material 5 [file 12870_2024_4821_MOESM5_ESM.docx]

Supplementary Table 1 The primers information in this study

| Gene | Orward sequence (5'-3') | Reverse sequence (5'-3') | PCR Products (bp) |
| --- | --- | --- | --- |
| ACT7 | CCAGGAATTGCTGACCGTAT | GTTGGAAGGTGCTGAGGGA | 142 |
| *4CL* (LOC110730923) | TTCAGGTTCCACCAGCAGAG | AAGGCGGCTTGAAGGATG | 106 |
| *C3’H* (LOC110717575) | TGCTAAAGCCAAGTCTCAAAAC | CCAAAGAAGCCCAATCACG | 115 |
| *CHI* (LOC110704458) | CAGCACCTGTGGAAACAGTTAT | CTGCCAATCGGTCCCTTAC | 102 |
| *CHS* (110724467) | CAAGTCGAGGCCAAACTAGG | AAACCCGAACAAAACACCC | 186 |
| *CYP75B1* (LOC110700687) | ATGGACCGACCCACTAGAGTT | CCCTGCCCCAAATGGAATA | 109 |
| *F3H* (LOC110724781) | CGAAGGGTGGAGGAATGTG | GAGCCCAAGAGTAAGGTCAGG | 193 |
| *FG3* (LOC110719441) | TCCACCGCCAACAACTCC | CAAACTCTTTCCACCTTCCGT | 148 |
| *HCT* (LOC110713661) | TAGCCAAGGCAGGCGATGT | CGGGTTGAGATTCAAGGTAGTC | 129 |
| *PAL* (LOC110724960) | AGGATGTGAACTCCCTCGGTC | TCTGCATACTTGGCTTACGGT | 173 |
| *CYP73A* (LOC110684642) | TGCCCACTGGAAGAACCC | CCAAACGACCAATAGTAATGCC | 173 |
