# Supplementary material for: Integrated transcriptomic and metabolomic analyses reveals anthocyanin biosynthesis in leaf coloration of quinoa (Chenopodium quinoa Willd.)
Source: BMC Plant Biol. 2024 Mar 20;24:203. doi: 10.1186/s12870-024-04821-2 (PMC10953167; doi:10.1186/s12870-024-04821-2)
Supplement: Supplementary file 6 — Supplementary Material 6 [file 12870_2024_4821_MOESM6_ESM.docx]

Supplementary Table 3 The number of total metabolites

| group | up | down | sum |
| --- | --- | --- | --- |
| N1 vs N2 | 381 | 1505 | 1712 |
| N1 vs N3 | 741 | 1613 | 1553 |
| N2 vs N3 | 808 | 473 | 1878 |
| F1 vs F2 | 940 | 1290 | 1886 |
| F1 vs F3 | 1149 | 1472 | 2354 |
| F2 vs F3 | 1254 | 1151 | 1281 |
| N1 vs F1 | 837 | 875 | 2230 |
| N2 vs F2 | 1054 | 499 | 2621 |
| N3 vs F3 | 1175 | 703 | 2405 |
