# Supplementary material for: Integrated transcriptomic and metabolomic analyses reveals anthocyanin biosynthesis in leaf coloration of quinoa (Chenopodium quinoa Willd.)
Source: BMC Plant Biol. 2024 Mar 20;24:203. doi: 10.1186/s12870-024-04821-2 (PMC10953167; doi:10.1186/s12870-024-04821-2)
Supplement: Supplementary file 7 — Supplementary Material 7 [file 12870_2024_4821_MOESM7_ESM.docx]

Supplementary Table 2. Analysis of transcriptomic sequencing quality

| sample | Raw Reads | Clean data (bp) | N (%) | Q20% | Q30% | GC (%) |
| --- | --- | --- | --- | --- | --- | --- |
| N1 | 8587572800 | 8521089852 | 0% | 98.17% | 94.50% | 45.38% |
| N2 | 8737575700 | 8665021391 | 0% | 97.96% | 94.04% | 45.66% |
| N3 | 7028937300 | 6972622440 | 0% | 97.52% | 92.96% | 45.15% |
| F1 | 6908289000 | 6853592286 | 0% | 97.68% | 93.37% | 46.18% |
| F2 | 7840968600 | 7787675930 | 0% | 98.02% | 94.15% | 45.62% |
| F3 | 8860698200 | 8796222315 | 0% | 97.84% | 93.73% | 45.16% |
