# Supplementary material for: Integrated transcriptomic and metabolomic analyses reveals anthocyanin biosynthesis in leaf coloration of quinoa (Chenopodium quinoa Willd.)
Source: BMC Plant Biol. 2024 Mar 20;24:203. doi: 10.1186/s12870-024-04821-2 (PMC10953167; doi:10.1186/s12870-024-04821-2)
Supplement: Supplementary file 8 — Supplementary Material 8 [file 12870_2024_4821_MOESM8_ESM.docx]

Supplementary Table 4. FPKM values of transcription factors in each sample

| TF family | Gene name | Gene ID | N1 | N2 | N3 | F1 | F2 | F3 |
| --- | --- | --- | --- | --- | --- | --- | --- | --- |
| bHLH | MYC2 | LOC110730184 | 295.61 | 192.72 | 109.71 | 64.59 | 226.65 | 34.29 |
|  |  | LOC110732543 | 124.63 | 66.28 | 37.12 | 18.19 | 100.99 | 8.33 |
|  | bHLH14 | LOC110732803 | 2.68 | 4.59 | 9.27 | 3.9 | 9.29 | 4.07 |
|  |  | LOC110731636 | 1.81 | 1.69 | 1.02 | 6.23 | 4.73 | 0.26 |
|  |  | LOC110714364 | 0.81 | 0.94 | 0.47 | 8.16 | 1.35 | 1.50 |
| bZIP | HY5 | LOC110704486 | 15.40 | 17.28 | 7.15 | 10.00 | 6.12 | 22.26 |
|  |  | LOC110723164 | 16.71 | 22.41 | 7.46 | 9.42 | 4.05 | 25.51 |
|  | TGA | LOC110682168 | 14.23 | 30.90 | 28.00 | 17.05 | 27.68 | 40.03 |
| WRKY | WRKY24 | LOC110684199 | 211.06 | 190.1 | 129.80 | 30.1 | 184.48 | 21.81 |
|  |  | LOC110684669 | 161.95 | 172.14 | 142.52 | 70.67 | 156.59 | 19.85 |
|  |  | LOC110698105 | 9.56 | 23.79 | 42.28 | 10.49 | 57.38 | 7.47 |
|  |  | LOC110730454 | 11.47 | 27.42 | 47.98 | 9.85 | 57.75 | 7.95 |
|  | WRKY46 | LOC110701898 | 4.67 | 7.41 | 10.54 | 6.95 | 6.54 | 12.57 |
